# Study on* Yang-Xu* Using Body Constitution Questionnaire and Blood Variables in Healthy Volunteers

**DOI:** 10.1155/2016/9437382

**Published:** 2016-05-31

**Authors:** Hong-Jhang Chen, Yii-Jeng Lin, Pei-Chen Wu, Wei-Hsiang Hsu, Wan-Chung Hu, Trong-Neng Wu, Fang-Pey Chen, Yun-Lian Lin

**Affiliations:** ^1^National Research Institute of Chinese Medicine, Taipei 112, Taiwan; ^2^Taipei Veterans General Hospital, Center for Traditional Medicine, Taipei 112, Taiwan; ^3^Biostatistics Center, Taipei Medical University, Taipei 110, Taiwan; ^4^Department of Chinese Pharmaceutical Sciences and Chinese Medicine Resources, China Medical University, Taichung 404, Taiwan; ^5^Department of Nursing, Hung Kuang University, Taichung 433, Taiwan; ^6^Institute of Traditional Medicine, School of Medicine, National Yang-Ming University, Taipei 112, Taiwan; ^7^School of Pharmacy, National Taiwan University, Taipei 100, Taiwan

## Abstract

Traditional Chinese medicine (TCM) formulates treatment according to body constitution (BC) differentiation. Different constitutions have specific metabolic characteristics and different susceptibility to certain diseases. This study aimed to assess the* Yang-Xu* constitution using a body constitution questionnaire (BCQ) and clinical blood variables. A BCQ was employed to assess the clinical manifestation of* Yang-Xu*. The logistic regression model was conducted to explore the relationship between BC scores and biomarkers. Leave-one-out cross-validation (LOOCV) and K-fold cross-validation were performed to evaluate the accuracy of a predictive model in practice. Decision trees (DTs) were conducted to determine the possible relationships between blood biomarkers and BC scores. According to the BCQ analysis, 49% participants without any BC were classified as healthy subjects. Among them, 130 samples were selected for further analysis and divided into two groups. One group comprised healthy subjects without any BC (68%), while subjects of the other group, named as the sub-healthy group, had three BCs (32%). Six biomarkers, CRE, TSH, HB, MONO, RBC, and LH, were found to have the greatest impact on BCQ outcomes in* Yang-Xu* subjects. This study indicated significant biochemical differences in* Yang-Xu* subjects, which may provide a connection between blood variables and the* Yang-Xu* BC.

## 1. Introduction

Traditional Chinese medicine (TCM) formulates treatment under the guidance of the holistic concept according to constitution differentiation (Bian Zheng Lun Zhi; Zheng classification) [1]. Thus, treatment for the same patient can vary over time while the same disease in different patients can be treated differently. Hence, Zheng classification in TCM adopts a personalized approach to treatment of diseases. Zheng diagnosis is the key process to distinguish a person in a state of sub-health, subdisease, or predisease which is different from the “normal” situation and to differentiate one disease from another as well as the choice of the methods for the treatment [2–4]. However, constitution differentiation in TCM is made by looking, listening and smelling, asking, and touching. Till now, people still challenge the fact that the Chinese clinical practice depends only on observation, knowledge, and clinical experiences of Chinese medical doctors (CMD), which lacks scientific basis [5].

*Yin-Yang* are two opposite, complementary, and exchangeable aspects of nature. The classification of* Yin-Yang* is usually the first step of Zheng classification in TCM.* Yin* mainly refers to the organs and* Yang* to the functions. In healthy status,* Yin-Yang* is in balance. Disease occurs after a disturbance in* Yin-Yang* or disharmony in the organs caused by pathogenic or climatic and environmental factors. Treatment aims to expel or suppress the causes and restore balance [1]. Body constitution (BC) has been the fundamental theory in clinical TCM practice for thousands of years and has been employed to differentiate an individual or a patient in the states of sub-health, subdisease, or predisease [3, 6, 7]. The special state usually determines a person's physiological reaction and susceptibility to some pathogenic factors as well as tendency towards pathogenic modes [6]. According to the TCM theory and the relationships of biomarkers observed in patients, a BC questionnaire (BCQ) was developed to bridge the gap between the personalized medical approach of TCM and modern clinical research methodology [8–15].

The developed BCQ consists of 44 items, which are divided into three imbalanced BC scales, namely,* Yin-Xu*,* Yang-Xu*, and* Stasis* [8, 10, 12]. A pilot test and clinical studies on its reliability and validity were carried out [9]. Results revealed that five domains with 19 items in the BCQ had high sensitivity and specificity [11, 14]. However, some of the 19 items had poorer validity and reliability and the composite questions of some extracted factors were less consistent, thus influencing the significance level of more than one imbalanced BC type and causing individual bias of CMD in BC diagnosis [9]. The system biology-based approach, especially metabolomics, has been applied to TCM constitution differentiation, which aims to make TCM practice more scientific and evidence-based [5, 16–18]. This study evaluated the BCQ using the parameters in 26 clinical blood variables from healthy volunteers and established the connection between* Yang-Xu* and BCQ using these blood clinical variables.

## 2. Materials and Methods

### 2.1. Subjects

Healthy volunteers of mostly young subjects (age ranging from 20 to 39 years) were recruited between March 2013 and June 2013 from the Taipei Veterans General Hospital (Taipei, Taiwan). This study complied with the Declaration of Helsinki and was approved by the Institutional Review Board (IRB, number 2012-10-015B). A Chinese medical doctor, Yii-Jeng Lin (male aged 39), with a degree in medicine, a license as a Chinese physician, and more than 6 years of clinical experience, explained the details of the study to the subjects and solicited written informed consent from each subject. Subjects who had taken any medicine, dietary supplement, or Chinese medicine within the previous month were excluded from the study. A total of 192 subjects (92 male and 100 female) were recruited. The characteristics of the participants are shown in Table 1.

### 2.2. Design and Study Population

Three types of data were collected including the BCQ responses, biomarkers, and clinical records of a Chinese medical doctor (CMD). The BC was assessed from the self-reported BCQ responses and by the CMD. Clinical manifestations of* Yang-Xu*,* Yin-Xu*, and* Stasis* were evaluated using the BCQ [8–12], while the CMD reviewed the results of each indicator of* Yang-Xu*,* Yin-Xu*, and* Stasis*. This study used 26 biomarkers as the BCQ indicators.

People with simultaneous clinical manifestations of* Yang-Xu*,* Yin-Xu*, and* Stasis* according to the BCQ responses were classified as abnormal. The CMD also deemed subjects with two or more clinical manifestations among* Yang-Xu*,* Yin-Xu*, and* Stasis* as abnormal. People without any constitutions of* Yang-Xu*,* Yin-Xu*, and* Stasis* were taken as normal. Finally, 130 participants including 88 normal individuals and 42 abnormal subjects were selected according to the BCQ results.

### 2.3. Blood Biochemical Analysis

This study hypothesized that the underlying predisease or sub-healthy conditions might modify BC. Such conditions include poor nutrition, sub-healthy endocrine problems, and minimal inflammation. In view of this, the nutritional (ALB, HDC, and LDL), endocrine (TSH, LH, Glu-AC, Cortisol, and DHEAS), inflammation status (HSCRP, ALT, and AST) and renal function (BUN, CRE) were evaluated at routine check-up. Routine clinical chemical analysis of fasting blood samples was conducted by Union Clinical Laboratory, which has been accredited by the College of American Pathologists Laboratory Accreditation Program (LAP number 6979606). The 26 biomarkers were albumin (ALB), alanine transaminase (ALT), aspartate transaminase (AST), blood urea nitrogen (BUN), creatinine (CRE), cortisol (CORS), fasting blood glucose (Glu-AC), high-density lipoprotein cholesterol (HDLC), low-density lipoprotein cholesterol (LDLC), luteinizing hormone (LH), thyroid-stimulating hormone (TSH), high-reactive C-reactive protein (HSCRP), dehydroepiandrosterone (DHEAS), and red blood cell (RBC) count, white blood cell (WBC) count, hemoglobin (HB), hematocrit (HCT), mean corpuscular volume (MCV), mean corpuscular hemoglobin (MCH), mean corpuscular hemoglobin concentration (MCHC), platelet (PLT), neutrophil (NEUT), lymphocyte (LYMPH), monocyte (MONO), eosinophil (EO), and basophil (BASO).

### 2.4. Body Constitution Questionnaire (BCQ)

The body constitution questionnaire (BCQ) was provided by Professor Yi-Chang Su, Ph.D., School of Chinese Medicine, China Medical University, Taichung, Taiwan. The physiological states of* Yang-Xu* [8, 9],* Yin-Xu* [10, 11], and* Stasis* were assessed using the BCQ [12]. The questionnaire consists of 44 items to be ranked with a 5-point Liker scale (from 1 (never happened) to 5 (always happened)) with a total score ranging from 44 to 220 (total score ranging from 55 to 173 found in this study). All items were characterized into three different BC scales, including 19 items in* Yang* deficiency [8], 19 items in* Yin* deficiency [10], and 16 items in* Stasis* [12]. Some items belong to more than one scale. For* Yang* deficiency (total score ranging from 19 to 95), a score exceeding 31 indicated* Yang-Xu* BC; for* Yin* deficiency (total score ranging from 19 to 95), a score exceeding 30 indicated* Yin-Xu* BC; and for* Stasis* (total score ranging from 16 to 80), a score exceeding 27 indicated* Stasis* BC. The higher the score, the greater the deviation of the BC. In a previous research, the BCQ had Cronbach's confidence *α* ranging from 0.55 to 0.88, with the intraclass correlation coefficients (ICC) exceeding 0.7 [8–10].

### 2.5. Chinese Medical Doctor (CMD)

In this study, The Chinese medical doctor, Dr. Yii-Jeng Lin, was responsible for making the diagnosis according to Chinese medicine theory and blinded to BCQ results. The major method of diagnosis was to take the patient's pulse caused by heartbeat.* Yang-Xu* is characterized by slow, weak, and faint pulses;* Yin-Xu* rapid and wiry pulses in float position and weak pulses in deep position; and* Stasis* sluggish pulses as well as asking about subjects' background. Each subject gave a score of 1 to 4 in each BC. The higher score indicated a higher level of the BC.

### 2.6. Logistic Regression Analysis

A logistic regression model was conducted to explore the relationship between BC scores and blood biomarkers [19, 20]. The response variable is binary and defines whether the participants are normal or abnormal. Logistic regression requires the variables to be independent of each other. Thus, the predictors in this study are 25 biomarkers only, with BASO, which is the linear combination of NEUT, LYMP, MONO, and EO excluded. Gender is one of the vital factors. With gender taken into account, the assumed logistic models are divided into two parts, part A and part B, as follows.

*Part A*. Pooled logistic regression model is

(1) $$\begin{array}{c} \log it\left(\;P_{i}\;\right)=\beta_{0}+\beta_{g}G+\beta_{1}X_{1}+\cdots +\beta_{26}X_{26}+\varepsilon . \end{array}$$

*Part B*. Separated logistic regression model is as follows:

 Female is
  (2) log⁡itPi=β0,0+β0,1X1+β0,2X2+⋯+β0,26X26+ε0.
 Male is
  (3) log⁡itPi=β1,0+β1,1X1+β1,2X2+⋯+β1,26X26+ε1.

### 2.7. Cross-Validation

Cross-validation (CV) is a better model evaluation method which is mainly used to assess how accurate a predictive model will perform in practice. Leave-one-out cross-validation (LOOCV) and *K*-fold cross-validation were conducted to examine the performance of the logistic regression model in this study [19].

### 2.8. Decision Trees

Decision trees were conducted to mine the possible relationship between 26 biomarkers and BC scores. Unlike the logistic regression model, a decision tree could suggest cut-off points when treating all factors as categorical variables and make a decision in the end. The deviance is employed to prune the full tree into a smaller tree without losing accuracy. Thus, the final model is formed [19–21].

## 3. Results

### 3.1. Blood Biochemical Analysis in Healthy Volunteers

A total of 192 healthy subjects volunteered to participate in this study. The characteristics of the subjects are summarized in Table 1. Both average BCQ scores and CMD diagnoses showed normal BC in all subjects. Furthermore, the blood biochemical analysis results were all within normal range (Table 2). Taken together, these results revealed that all subjects are in healthy condition.

### 3.2. BCQ Responses from Subjects

All subjects completed the questionnaire without any missing value. The BCQ score was 29.4 ± 8.1 in male* Yang-Xu* subjects and 32.2 ± 7.5 in female* Yang-Xu* subjects. The CMD score was 2.0 ± 0.9 in male and 1.9 ± 0.8 in female* Yang-Xu* subjects, respectively (Table 1).

Among them, 49% without any BC were classified as healthy subjects, and 39% have more than one BC (Table 3). The detailed BCQ classification is shown in Supplementary Table S1 (in Supplementary Material available online at http://dx.doi.org/10.1155/2016/9437382). For further analysis, more specific data, including those “without any BC” which represented the healthy subjects and those “with all three BCs” which represented the sub-healthy subjects, were extracted.  Table 4 shows the analysis results of 130 subjects separated into the healthy group (68%) and the sub-healthy group (32%).

### 3.3. Correlations between Clinical Blood Biomarkers and BC Scores

The logistic regression model was further conducted to explore the relationship between the BC scores and clinical blood biomarkers. The pooled logistic regression model assumed that the errors of male and female were the same and gender affected only the interception of the model, that is, *β*_*g*_. On the contrary, the separated logistic regression model was more flexible in estimating errors, *ε*_0_ and *ε*_1_, and the coefficients of predictors could vary. The pooled model was separated by gender; the model for male subjects was referred to as Model 1 while that for female subjects was referred to as Model 2. The error rate of the separated model was about 11.5%, which was less than that of the pooled model, about 23%. BC scores showed different trends for male and female subjects. Female had higher* Yang-Xu* and BC scores than male. Thus, the use of the separated logistic regression model was much reasonable than that of the pooled one.

Originally, the probability of participants in normal, *P*, was 77.8%. In other words, without using any model, the worst accuracy could be 77.8%. The BCQ male model (Model 1) gave a probability of correctly indicating a participant's status by taking the BCQ into consideration, $\hat{P}=90.3\%$. According to binominal distribution, the *P* value is 0.0049, which was less than the alpha level at 0.05. Hence, it can be concluded that Model 1 can significantly reduce the error rate when the logistic regression model was used for prediction. Same as Model 1, Model 2 (the female model) showed a *P* of 55.2% and $\hat{P}$ of 86.2%. *P* value of about 0.0000 at 0.05 alpha level represented that Model 2 can also significantly reduce the error rate when using the logistic regression model for prediction.

When using LOOCV and *K*-fold cross-validation to evaluate the performance of the aforementioned logistic regression models, both methods showed that the error rate went up to around 0.41 with standard deviation of 0.11. This means that the error rate in this study is high. Hence, although logistic models can explain the relationship between BC scores and biomarkers, the prediction is not as good as expected. Hence, a decision tree is proposed to solve the problem.

**Figure 1:**
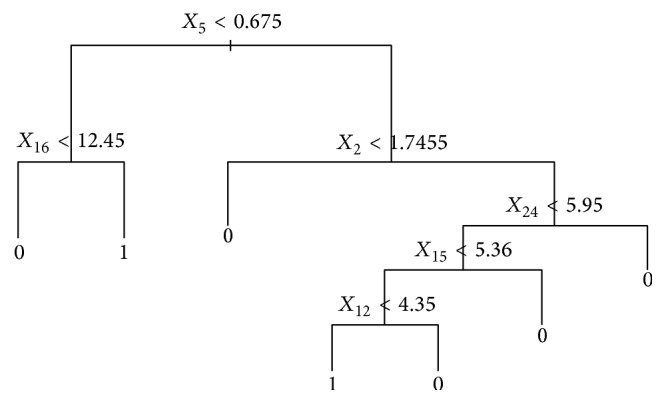
Final model of BCQ.

Figure  S1, a* BCQ decision tree*, shows the full model. Obviously, some leaves are redundant. Deviance was introduced to prune the full tree. After choosing tree size four, Figure  S2 shows that it maintains the relative high accuracy. Therefore, the final model is shown as in Figure 1. This model reveals clearly that six biomarkers, namely, CRE, TSH, HB, MONO, RBC, and LH, have the greatest impact on or closest relationship with the BCQ scores.

## 4. Discussion

A total of 192 subjects (92 male and 100 female) were recruited by the Center for Traditional Medicine of Taipei Veterans General Hospital. This pilot study was approved by the Institutional Review Board, and only participants aged between 20 and 39 years were included to avoid complicated parameters, such as aging and chronic inflammation. To evaluate the difference between sub-healthy status and healthy status, 88 participants without any BC were defined as healthy individuals, and 42 participants with three BCs were defined as sub-healthy subjects. BC in TCM represents the physical and physiological attributes of an individual. Different constitutions have specific metabolic characteristics and different susceptibility to certain diseases [2–4]. It is possible for coexistence of multiple imbalanced BCs in a subject, which is consistent with the Chinese medicine theories. In this study, six blood biomarkers, namely, CRE, TSH, LH, RBC, HB, and MONO, were found to have the greatest impact on BCQ outcomes in* Yang-Xu* subjects.

In* Yang-Xu* subjects classified according to BCQ, the results showed normal CRE, TSH, RBC, and HB levels but all lower than those of non-*Yang-Xu* subjects, while LH and MONO levels of* Yang-Xu* subjects are higher than those of non-*Yang-Xu* subjects. It is interesting that the six blood biomarkers, TSH, CRE, and LH, which are all regulated by estrogen, showed different levels in* Yang-Xu* female and male subjects (Supplementary Table S2).

*Yang-Xu* constitution implies a diminishing energy level in the physiological functioning of body. These individuals may experience symptoms including fatigue, nausea, dizziness, nasal congestion and allergy, mood swings, shortness of breath, chills, loose stool, and large volume of urine. Moreover, some diseases are significantly positively correlated with* Yang-Xu* [8–13]. Creatinine (CRE) is related to the mass of skeletal muscle and daily protein intake, as well as the waste created from the body's use of creatine, which is an amino acid found naturally in food sources, such as fish and meat. Assimilated creatine could be converted into creatine phosphate or phosphocreatine, which is used for energy. Additionally, phosphocreatine is also converted into ATP, or adenosine triphosphate, a significant source of physical and mental energy [22, 23]. Lower creatine levels were associated with fatigue, poor exercise capacity, muscle atrophy, and neuromuscular deficiencies [24, 25].

Thyroid-stimulating hormone (TSH) released by the pituitary in the brain informs the thyroid gland to secrete thyroxin (T4), which must then be converted into the active thyroid hormone, triiodothyronine (T3). Pituitary dysfunction in chronic fatigue syndrome may have a variety of causes, including viruses, bacteria, stress, toxins, inflammation, and mitochondria dysfunction. These problems could result in low normal TSH levels along with low normal T4 and T3 levels. Many studies also found that compared with the normal control group, the kidney-*Yang-Xu* group expressed lower levels of TSH, T3, and T4 [26, 27]. In addition, low normal TSH levels could cause fatigue, depression, and difficulty in losing weight [28, 29]. Hemoglobin (HB) carries oxygen, which is the main component of red blood cells. Lower HB level may result in chronic fatigue and poor concentration. Lower hemoglobin count may be associated with disease or any condition that causes the blood to have very few red blood cells (RBC). Common causes of anemia (low HB) include thalassemia, iron-deficiency anemia, and vitamin B12 deficiency. It is correlated directly with serum HB level. Symptoms of anemia are general malaise, dizziness, poor concentration, and even dyspnea on exertion [30, 31]. MONO is related to inflammation. Recent studies also indicated that values of host reactive cytokines of TNF-*α*, IL-6, IL-8, IL-10, and IL-18 were all significantly higher in* Yang-Xu* subjects [32]. These cytokines are produced by leukocytes and play a key role in regulating the activation of immune responses for hematopoiesis [33, 34]. The interleukin-family is involved in regulating the growth of blood cells and lymphoid cells, the development of bone-marrow cells, and the differentiation of T cells, B cells, and hematopoietic cells.

In data transformation, we rewrote the BCQ-model with the subjects having three BCs,* Yang-Xu*,* Yin-Xu*, and* Stasis,* from the results of BCQ. The subjects without any BC are denoted as normal. Thus, 32.29% of participants are eliminated. In this case, the proposed model can accurately represent the situation among those participants, almost 40% of relationship between biomarkers and BC scores.

This study used biochemical parameters to access* Yang-Xu* constitution for bridging the gap between Chinese medicine theory and BCQ. A more convincing digital parameter for personalized diagnosis remains to be performed with a larger sample size. To our knowledge, this study is the first report of using clinical blood variables to investigate* Yang-Xu* classified by the BCQ.

## Supplementary Material

Supplementary Table S1. A detailed classification of BCQ.
Supplementary Table S2. Comparison of the selected parameters of *Yang-Xu* vs. Non-*Yang-Xu* subjects in males and females (I); *Yang-Xu* vs. Non-*Yang-Xu* subjects by BCQ (II).
Supplementary Figure S1. The full model of BCQ.
Supplementary Figure S2. Relative high accuracy when tree size four is chosen.

## Figures and Tables

**Table 1:** Characteristics of 192 healthy young volunteers.

	Male	Female	Total
Participants	92	100	192
Age (year, mean ± SD)^a^	27.3 ± 4.5	26.4 ± 4.7	26.8 ± 4.6
BMI	23.3 ± 3.6	21.6 ± 3.5	22.5 ± 3.6
Education			
High school	4	5	9
Bachelor's degree	59	63	122
Master's degree	29	32	61
Occupation			
Student	43	57	100
Salaryman	32	27	59
Unemployment	9	6	15
Refused to answer	8	10	18
BCQ score^b^			
*Yang-Xu*	29.4 ± 8.1	32.2 ± 7.5	30.8 ± 7.9
*Ying-Xu*	29.0 ± 8.1	31.8 ± 7.1	30.5 ± 7.7
*Stasis*	24.3 ± 7.0	26.6 ± 7.7	25.5 ± 7.4
CMD score^c^			
*Yang-Xu*	2.0 ± 0.9	1.9 ± 0.8	1.9 ± 0.8
*Ying-Xu*	1.6 ± 0.7	1.6 ± 0.7	1.6 ± 0.7
*Stasis*	1.7 ± 0.7	1.8 ± 0.7	1.7 ± 0.7

^a^For male subjects, the age range is 20–38; for female subjects, the age range is 20–39.

^b^Score of body constitution questionnaire (BCQ) is as follows: in *Yang-Xu*, the total score range is 19–95; in *Yin-Xu*, the total score range is 19–95; in *Stasis*, the total score range is 16–80.

^c^Score of Chinese medical doctor (CMD) for each body constitution (BC) (*Yang-Xu*, *Yin-Xu*, or *Stasis*), the total score range is 1–4.

**Table 2:** Blood chemical analysis of subjects.

	Unit	Mean ± SD	
		Male	Female
ALB	g/dL	5.0 ± 0.2	4.8 ± 0.2
ALT	U/L	27 ± 18	19 ± 12
AST	U/L	24 ± 7	22 ± 6
BUN	mg/dL	13.5 ± 3.5	10.8 ± 2.5
CRE	mg/dL	0.95 ± 0.1	0.69 ± 0.09
CORS	*μ*g/dL	14.4 ± 4.6	12.8 ± 4.6
Glu-Ac	mg/dL	90 ± 6	87 ± 10
HDLC	mg/dL	56 ± 13	63 ± 13
LDLC	mg/dL	106 ± 25	96 ± 23
LH	mIU/mL	4.0 ± 1.7	7.1 ± 5.1
TSH	*μ*IU/mL	1.873 ± 0.749	1.875 ± 1.223
HSCRP	*μ*g/dL	0.119 ± 0.327	0.087 ± 0.253
DHEAS	mg/dL	326 ± 104	244 ± 92
RBC	10^6^/*μ*L	5.3 ± 0.4	4.69 ± 0.47
WBC	10^3^/*μ*L	6.14 ± 1.45	6.1 ± 1.6
HB	g/dL	16 ± 1	13 ± 1
HCT	%	46.1 ± 2.9	40.2 ± 2.9
MCV	fL	87.4 ± 2.9	86.3 ± 8.6
MCH	pg	29.7 ± 2.6	28.5 ± 3.4
MCHC	g/dL	33.9 ± 1.2	33.0 ± 1.0
PLT	10^3^/*μ*L	242 ± 55	268 ± 49
NEUT	%	55.6 ± 7.4	57.1 ± 8.1
LYMPH	%	34.9 ± 7.2	34.5 ± 8.0
MONO	%	5.8 ± 1.6	5.3 ± 1.3
EO	%	3.1 ± 1.8	2.5 ± 2.1
BASO	%	0.5 ± 0.3	0.5 ± 0.3

**Table 3:** Distribution of body constitution types classified by BCQ.

Classification of BCQ		Male	Female	Total	
Healthy	Without any body constitution	58	36	94	(49.0%)

Single body constitution	*Yang-Xu*	4	6	10	(5.2%)
	*Yin-Xu*	3	8	11	(5.7%)
	*Stasis*	1	1	2	(1.0%)

Complicated body constitution	*Yang-Xu* and *Yin-Xu*	7	13	20	(10.4%)
	*Yang-Xu *and* Stasis*	1	6	7	(3.6%)
	*Yin-Xu *and* Stasis*	1	5	6	(3.1%)
	*Yang-Xu *and* Yin-Xu *and* Stasis*	17	25	42	(21.9%)

Total		92	100	192	(100%)

**Table 4:** More specific results of BCQ.

Classification	Male	Female	Total
	BCQ^a^		
*N*	73	57	130
*Healthy*	56 (77%)	32 (56%)	88 (68%)
*Sub-healthy*	17 (23%)	25 (44%)	42 (32%)

^a^Healthy means* 000*; sub-healthy means *111*.
