# Supplementary material for: Study on Yang-Xu Using Body Constitution Questionnaire and Blood Variables in Healthy Volunteers
Source: Evid Based Complement Alternat Med. 2016 May 31;2016:9437382. doi: 10.1155/2016/9437382 (PMC4906219; doi:10.1155/2016/9437382)
Supplement: Supplementary file 1 — Supplementary Table S1. A detailed classification of BCQ. Supplementary Table S2. Comparison of the selected parameters of Yang-Xu vs. Non-Yang-Xu subjects in males and females (I); Yang-Xu vs. Non-Yang-Xu subjects by BCQ (II). Supplementary Figure S1. The full model of BCQ. Supplementary Figure S2. Relative high accuracy when tree size four is chosen. [file 9437382.f1.pdf]

Supplementary Table S1

<table>
<tr><td rowspan="9">BCQ</td><td>Yang-Xu</td><td>Yin-Xu</td><td>Stasis</td><td>Mark</td><td>Stndrome</td><td>Male</td><td>Female</td><td>Total</td></tr>
<tr><td>0</td><td>0</td><td>0</td><td>0</td><td>Healthy</td><td>56</td><td>32</td><td>88</td></tr>
<tr><td>1</td><td>0</td><td>0</td><td colspan="2" rowspan="6">Excluded</td><td>4</td><td>4</td><td rowspan="6">62</td></tr>
<tr><td>0</td><td>1</td><td>0</td><td>6</td><td>11</td></tr>
<tr><td>0</td><td>0</td><td>1</td><td>0</td><td>2</td></tr>
<tr><td>1</td><td>1</td><td>0</td><td>7</td><td>13</td></tr>
<tr><td>1</td><td>0</td><td>1</td><td>1</td><td>6</td></tr>
<tr><td>0</td><td>1</td><td>1</td><td>1</td><td>7</td></tr>
<tr><td>1</td><td>1</td><td>1</td><td>1</td><td>Sub-Healthy</td><td>17</td><td>25</td><td>42</td></tr>
</table>

In *Yang-Xu* (The total score range is 19-95). *0* means score ≦31, *1* means score ≧32.
In *Yin-Xu* (The total score range is 19-95). *0* means score ≦30, *1* means score ≧31.
In *Stasis* (The total score range is 16-80). *0* means score ≦27, *1* means score ≧28.

Supplementary Table S2

Comparison of selected parameters of *Yang-Xu* male and female subjects (I); *Yang-Xu* vs. Non-*Yang-Xu* subjects classified by BCQ (II).

(I)

	Gender	TSH	CRE	LH	RBC	HB	MONO
***Yang-Xu* (vs. non-*Yang-Xu*)**	**Male**	↓	↑	↑	↓	↓	↑
	**Female**	↑	↓	↓	↓	↓	↑
	**total**	↓	↓	↑	↓	↓	↑

(II)

	TSH	CRE	LH	RBC	HB	MONO
***Yang-Xu* (vs. non-*Yang-Xu*)**	↓	↓	↑	↓	↓	↑

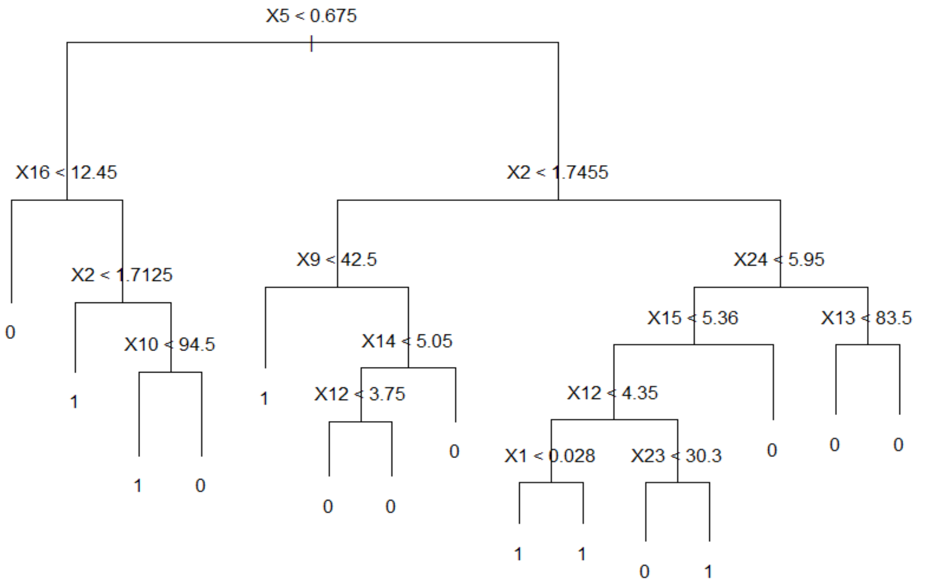

Supplementary Figure S1. Full model of BCQ. A BCQ decision tree shows the full model. Obviously, some leaves are redundant.

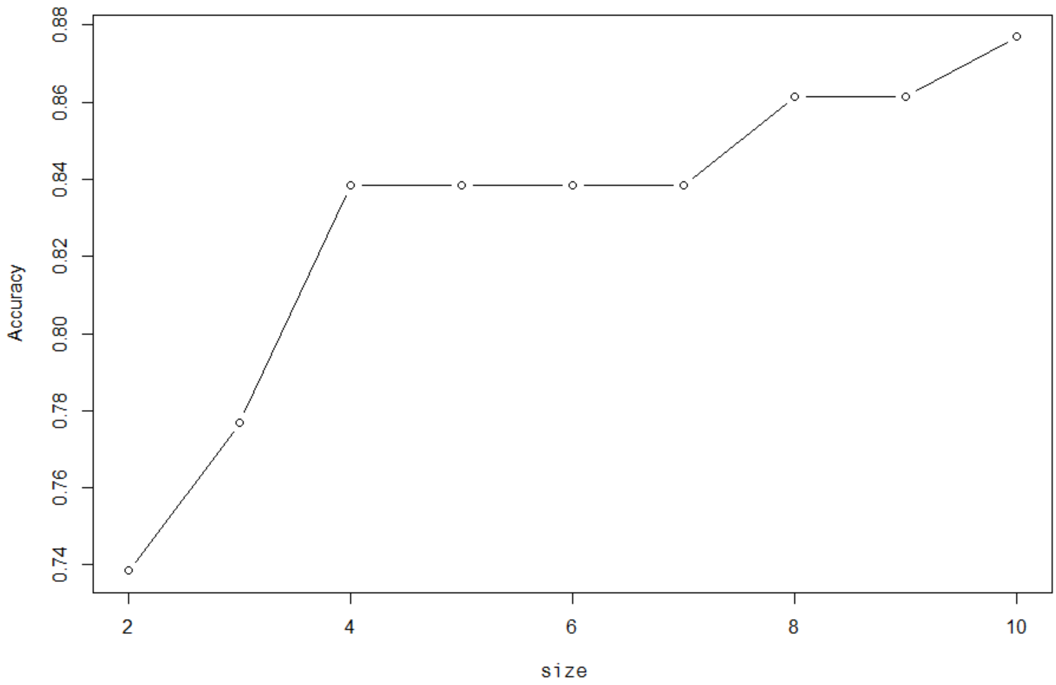

Supplementary Figure S2. Relative high accuracy when tree size four is chosen. A BCQ decision tree in Figure S1 shows some redundant leaves. Deviance was introduced to prune the full tree. After choosing tree size four, it maintained the relative high accuracy.
